# PTEN-mediated senescence of lung epithelial cells drives ventilator-induced pulmonary fibrosis

**DOI:** 10.7150/thno.117523

**Published:** 2025-07-25

**Authors:** Mengyu Li, Yu Wang, Zhiqiang Hu, Shiqian Huang, Pu Chen, Lin Chen, Jing Wu, Zhouyang Wu, Shanglong Yao, Yiyi Yang

**Affiliations:** 1 Department of Anesthesiology, Union Hospital, Tongji Medical College, Huazhong University of Science and Technology, Wuhan, 430022, China.; 2 Institute of Anesthesia and Critical Care Medicine, Union Hospital, Tongji Medical College, Huazhong University of Science and Technology, Wuhan, 430022, China.; 3 Key Laboratory of Anesthesiology and Resuscitation (Huazhong University of Science and Technology), Ministry of Education, China.; 4 Hubei Provincial Clinical Research Center for Anesthesiology, Wuhan 430022, China.

**Keywords:** mechanical ventilation-pulmonary fibrosis, epithelial senescence, phosphatase and tensin homolog, P53 signaling

## Abstract

Rationale: Mechanical ventilation (MV), a life-saving intervention for acute respiratory distress syndrome (ARDS), may exacerbate pulmonary fibrosis (PF) through unclear mechanisms. Although Phosphatase and Tensin homolog (PTEN) suppresses chronic PF, its role in MV-induced PF remains unknown. This study will determine whether PTEN mediates MV-PF via lung epithelial cell senescence.

**Methods:** Human lung epithelial cells exposed to hydrochloric acid (HCl) and mechanical stretch (48 hours) and a murine "two-hit" (HCl+MV) model (14-day observation) were used. PTEN's role was assessed via siRNA (*in vitro*) and knockout (*in vivo*). Single-cell transcriptomics analyzed senescence-associated secretory phenotype (SASP) and pathway enrichment. RG7388 (MDM2-P53 inhibitor) was administered to PTEN knockout mice to evaluate P53-mediated senescence.

**Results:** HCl+MV induced epithelial-mesenchymal transition (EMT) and fibrosis *in vitro* and *in vivo*. PTEN knockout or knockdown attenuated these effects. Single-cell profiling indicated PTEN's role in EMT and fibrosis via cell senescence pathways, particularly in epithelial cells exhibiting imbalances in the SASP scores. Furthermore, our experiments confirmed that senescence activation during fibrosis was reversed by PTEN inhibition. RG7388 treatment in PTEN knockout mice implicated P53-mediated senescence in PTEN's regulatory role.

**Conclusions:** Our study demonstrates that PTEN plays a pivotal role in MV-PF, by mediating pulmonary epithelial cell senescence. Future studies may focus on developing strategies to modulate PTEN activity and cell senescence to prevent or treat this devastating disease.

## Introduction

Acute lung injury/acute respiratory distress syndrome (ALI/ARDS) represents a life-threatening progressive disorder characterized by a triphasic disease progression consisting of exudative, proliferative, and fibrotic phases 1,2. The initial exudative phase (≤7 days post-injury) stems from endothelial dysfunction, while later stages feature collagen deposition and epithelial cells (ECs) damage leading to pulmonary fibrosis (PF), a form of interstitial lung disease (ILD) 3-5. This fibrotic transformation, marked by irreversible extracellular matrix (ECM) accumulation, impairs gas exchange and drives poor outcomes 3,6, underscoring the critical need for effective interventions. Mechanical ventilation (MV) remains a critical intervention for managing ARDS, yet it paradoxically contributes to ventilator-induced lung injury and accelerates PF development 7,8. Clinically, PF was detected in open-lung biopsies from 53% of patients ventilated ≥5 days 9. Prolonged ventilation (>12 days) was associated with a 64% incidence of PF and 57% mortality 10. Unlike chronic ILD (e.g., idiopathic pulmonary fibrosis [IPF]), which develops over years, MV-induced PF represents a rapidly progressive form of acute ILD driven by mechanical stress and resulting in accelerated ECM deposition.

Emerging evidence suggests that repetitive mechanical stress from cyclic alveolar inflation/deflation during MV initiates pathological cascades involving inflammatory infiltration and oxidative damage 11. Of particular interest is epithelial-mesenchymal transition (EMT), a reparative mechanism whereby ECs acquire mesenchymal phenotypes 12. While essential for wound healing, dysregulated EMT has been increasingly recognized as a driver of fibrotic remodeling in ventilator-associated lung injury 13,14. The molecular pathways linking MV to EMT activation remain poorly understood. Our previous work identified Piezo1 mechanoreceptors 15 and lipid mediators like Resolvin D1 16 as potential regulators of MV-induced EMT activation and fibrosis. Further investigation into these pathways may reveal therapeutic targets to mitigate ventilation-associated fibrotic complications and improve patient outcomes.

Phosphatase and tensin homolog deleted on chromosome 10 (PTEN), a recently characterized dual-specificity lipid/protein phosphatase, serves as a critical regulator of cellular growth, survival, and migration. Emerging evidence demonstrates its anti-fibrotic potential through negative regulation of the integrin-PI3K/Akt proliferative pathway, with documented therapeutic effects across multiple organ systems including hepatic 17 and renal fibrosis 18,19. PTEN functions as an antifibrotic mediator in chronic IPF by stabilizing the alveolar basement membrane and suppressing fibroblast proliferation, differentiation, and collagen secretion 20. Notably, the pathomechanisms underlying MV-associated pulmonary fibrosis (MV-PF) differ fundamentally from chronic IPF 20. Despite PTEN's established protective roles in chronic fibrosis models, its functional dynamics in this distinct mechano-inflammatory fibrotic paradigm remain unexplored.

Our investigation into MV-PF revealed an unexpected protective effect of PTEN depletion, which markedly attenuated cellular senescence in pulmonary EC and subsequently ameliorated EMT progression and fibrotic remodeling. Intriguingly, emerging experimental evidence also demonstrated that PTEN exhibits pro-fibrotic effects in cardiac fibrosis by driving M2 macrophage polarization 21. These paradoxical findings challenge the conventional understanding of PTEN as a known fibrosis suppressor. We therefore propose a novel regulatory paradigm: PTEN may exacerbate acute MV-PF through senescence potentiation in mechano-stressed epithelium. PTEN may serve as a potential "phase-specific regulator" of fibrogenesis, suppressing fibrosis in chronic microenvironments (e.g., IPF) while paradoxically accelerating acute fibrogenesis through senescence activation (e.g., MV-PF).

Recent preclinical studies have established a pathophysiologically-relevant "dual-hit" murine model (HCl aspiration-induced ARDS + MV) to investigate mechanotransduction-mediated EMT in MV-PF 22,23. This experimental paradigm uniquely recapitulates the clinical progression from aspiration-induced alveolar injury to ventilation-aggravated fibrogenesis. Through integrated single-cell transcriptomics and conditional PTEN knockout models, we systematically characterized the phase-specific mechanoregulatory function of PTEN, employing *in vivo* and ex vivo analyses to dissect its therapeutic targetability in acute mechanical injury contexts.

## Methods

### Cell culture and Si-PTEN transfection

Human lung bronchial EC (BEAS-2B) were purchased from the American Type Culture Collection (ATCC) and cultured according to the manufacturer's instructions. The cells were passaged at a 1:3 ratio every other day, with the culture medium changed every two days. All cell lines used in this study were authenticated by Short Tandem Repeat (STR) analysis and tested for mycoplasma contamination.

BEAS-2B cells were seeded in a six-well plate and cultured until the cell density reached approximately 40% for transfection. The siRNA lyophilized powder was formulated into a 20 μM solution, followed by the preparation of an siRNA-Lipo2000 complex (5 μl of Lipo2000, 150 μl of Opti-Minimal Essential Medium (MEM), and 5 μl of siRNA). The control wells received 2 ml of Opti-MEM, while the transfection wells initially received 1.69 ml of Opti-MEM, followed by the addition of 310 μl of the siRNA-Lipo2000 complex. The six-well plate was gently agitated to mix the medium thoroughly. The plate was then incubated in a 37°C cell culture incubator with CO_2_ for 72 hours. After incubation, total RNA was extracted from the cells, and the knockdown efficiency of PTEN was verified using real-time quantitative PCR.

### Mechanical stretch system and *in vitro* cell studies

HCl stimulation was used to mimic lung injury caused by aspiration of gastric contents in clinical patients, and the mechanical stretch system was used to simulate the cyclic stretching and contraction of cells, mimicking the use of ventilators in clinical treatment. Cells were seeded into both standard 6-well plates at densities of 4.0×10^5 cells/well. On the following day, once the cells reached a confluence of 40%, 2 ml of Dulbecco's Modified Eagle Medium (DMEM) with a pH of 4.0 was added. After a 30-minute treatment, the supernatant was immediately discarded, and the wells were gently washed twice with 2 ml of sterile PBS. Then, 2 ml of complete DMEM containing 10% serum was added, and the plates were placed in a 37°C CO_2_ incubator for subsequent stretching treatment.

The cell model was applied using the Flexcell FX500T cell strain loading system. The strain system, controlled by a vacuum pump, creates negative pressure on a collagen-coated elastic culture membrane, causing the attached cells to experience mechanical strain through expansion and contraction. The magnitude of the strain experienced by the cells is related to the degree of stretch applied to the culture membrane, known as the strain rate (%). In this experiment, a 20% strain rate was used, with a loading program controlled by FX-5000 computer software.

### Ethics statement for animal experiments

All animal experiments were conducted with strict adherence to the revised Institute of Laboratory Animal Resources, Commission on Life Sciences, National Research Council "Guide for the Care and Use of Laboratory Animals" National Academy Press, Washington, D.C. 1996, and protocols endorsed by the Ethics Committee of Tongji Medical College, Huazhong University of Science and Technology (IACUC Number: 3733), ensuring the highest standards of animal welfare and research integrity.

### Generation of conditional PTEN knockout mice

To generate male mice with tamoxifen-inducible PTEN deletion specifically in the lung EC (PTEN^CKO^), PTEN^F/F^ mice (PTEN^tm2.1Apat/^J; Jackson Laboratory) were crossed with Sftpc-Cre^ERT^ mice (Jackson Laboratory). Genotyping was undertaken by PCR using primers specific for Cre and PTEN. To knock out PTEN, 3-week-old PTEN^F/F^; Sftpc-Cre^ERT^ mice were given a tamoxifen suspension (0.1 ml of 10 mg/ml) (HY-Y1888, MCE, USA) by intraperitoneal injection for 7 d. Control groups consisted of age- and weight-matched PTEN^F/F^ mice. The efficacy of PTEN knockout in lung EC was validated through genotyping and confirmed by immunofluorescent double labeling, ensuring the precision of our conditional knockout model.

### Two-hit model of MV after acid aspiration-induced lung injury in mice

Mice were anesthetized by intraperitoneal injection of 2% pentobarbital. After induction of anesthesia, a 24G arterial catheter was used for tracheal intubation, with the needle tip cut to prevent damage to the oral cavity and trachea. Following successful intubation, 2 ml/kg of HCl with a pH of 1.2 was instilled into the trachea, and the chest was quickly compressed to induce coughing, ensuring the HCl diffused throughout the lungs to avoid localized inflammation. After extubation, mice were placed on an electric heating pad to recover and returned to their cages for continued housing. Twenty-four hours later, mice were re-anesthetized, intubated, and mechanically ventilated for 2 hours using a small animal ventilator with a respiratory rate of 120 breaths per minute, an inspiratory/expiratory ratio of 1:1, and a tidal volume of 3 ml/kg. After ventilation, mice were allowed to recover on a heating pad and then returned to their cages for a further 14 days of housing. Samples were collected on day 14 for analysis.

### Single cell transcriptome analysis

The 10x Genomics platform was utilized to perform single-cell transcriptome analysis. This technology encapsulates cells with beads carrying unique Cell Barcodes in droplets, allowing for the collection of cell-containing droplets and subsequent lysis within the droplets. This process links mRNA within the cells to the Cell Barcodes on the beads, forming Single Cell GEMs. Reverse transcription reactions are carried out within the droplets to construct cDNA libraries, which are then sequenced to determine the absolute quantity of each transcript molecule within individual cells, utilizing UMI and Cell Barcode information.

Quality control of the samples was performed using the 10x genomics official software Cell Ranger, which integrates the STAR software for aligning reads to the reference genome. This process provides quality control metrics such as the number of high-quality cells, genes, and genome alignment rates, allowing for the assessment of sample quality.

### Histology and scoring of lung injury

Lung tissue was stained with Hematoxylin and Eosin (HE) for histological examination of lung injury. The process involved fixing lung tissue in 4% paraformaldehyde for 24 hours, paraffin embedding, and sectioning at 5μm. Sections were deparaffinized and rehydrated through a series of xylene and ethanol washes, followed by staining with hematoxylin for nuclei and eosin for cytoplasm. After staining, sections were dehydrated, cleared, and mounted for microscopic examination and photography.

For Masson's trichrome staining to assess lung fibrosis, a similar deparaffinization and rehydration process was followed, with specific staining steps including hematoxylin, Masson's trichrome, phosphomolybdic acid, and aniline blue. After staining, sections were dehydrated, cleared, and mounted. Lung fibrosis was scored using the Ashcroft scale.

### Western blot (WB) assay

Use SDS-PAGE gels to separate equal amounts of protein lysate. Transfer the proteins to a nitrocellulose filter membrane (Millipore, USA). Incubate with primary antibody and secondary antibody sequentially. Then, the membranes were treated using Western Quick Block Kit (PS108, Epizyme Biotech, China) in dark. Eventually, the western blot bands were captured using BioRID ChemiDoc XRS+ (Bio-Rad, Hercules, CA, USA). Antibodies are listed in Supplementary Table S1.

### Imunofluorescence assay

Freshly prepared lung OCT sections or cell climbing films were incubated with 0.2% Triton X-100 in PBS for 15 min followed by 5% bovine serum albumin for 30 min at room temperature. The slides were then incubated with primary antibody respectively at 4°C overnight. After washing, the slides were incubated with FITC-labeled secondary antibody respectively for 60 min at room temperature. Slides were counterstained with DAPI, cover-slipped, and fluorescent images captured using a Nikon A1R Confocal Microscope (Tokyo, Japan). Antibodies are listed in Supplementary Table S1.

### Quantitative real-time PCR

Novozymes RNA Extraction Kit (Invitrogen, Carlsbad, CA, USA) was used to extract total RNA. Novozymes Reverse Transcription Kit with gDNA Eraser (RR047A, TAKARA, Shiga, Japan) was used to reverse transcribe total RNA to cDNA. The quantitative reverse transcription PCR analysis was performed using FastStart Universal SYBR Green Master (4913914001, Roche, Basel, Switzerland) and the StepOne Real-time PCR System (Applied Biosystems, Foster City, CA, USA). Using the 2-ΔΔCt method to calculate relative folding changes. The primer sequences are detailed in Table S2.

### Enzyme-linked-immunosorbent serologic assay (ELISA)

On day 14 post-modeling, bronchoalveolar lavage fluid (BALF) was collected for ELISA detection of TGF-β1, IL-1β, and IL-6. Lung tissue was also used for ELISA detection of Collagen-I and hydroxyproline. The assay methods were performed according to the manufacturer's instructions.

### Statistical analysis

The data are presented as the mean ± SED. Comparisons between two groups were performed using independent sample t tests. One-way ANOVA and the Bonferroni post hoc test were used to compare more than two groups. All statistical analyses were performed using Prism 9 (GraphPad Software, USA), and a *P* value<0.05 indicated statistical significance.

## Results

### MV exacerbated PF and EMT, downregulated PTEN expression

**Figure 1:**
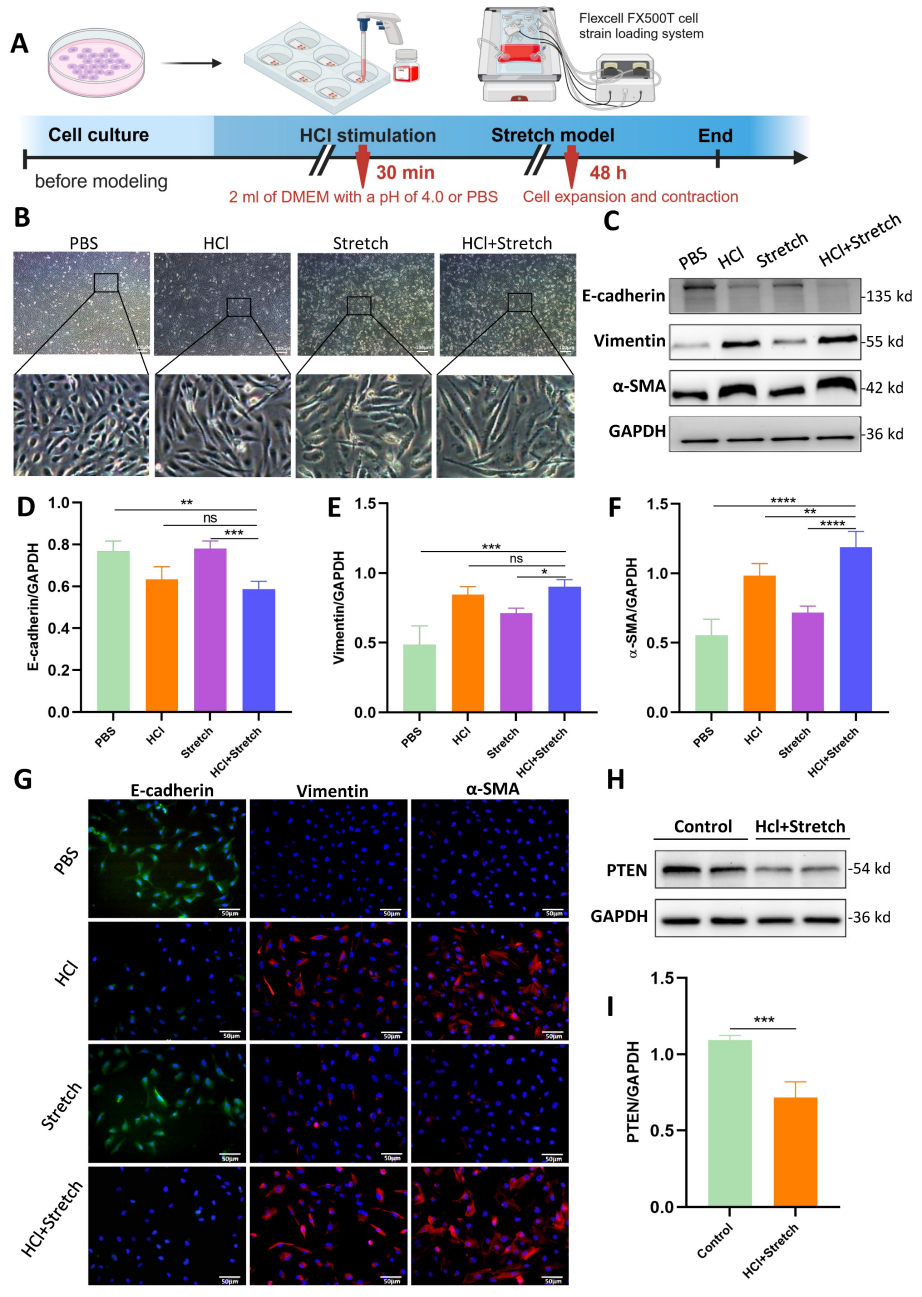
***In vitro* effects of HCl and mechanical stretch on cellular morphology and epithelial mesenchymal transition (EMT).** (A) Schematic of mechanical stretch and HCl injury model in BEAS-2B cells. Cells were cultured and subjected to HCl stimulation followed by mechanical stretch using the Flexcell FX500T cell strain loading system. (B) Representative phase-contrast images showing morphological changes (spindle shape, intercellular spacing) under different conditions: control (PBS), HCl stimulation, stretch, and HCl + stretch. (C) Western blot analysis of E-cadherin, Vimentin, and α-SMA protein expression in control (PBS), stretch, and HCl-treated cells with or without stretch. GAPDH served as a loading control. (D-F) Quantification of E-cadherin (epithelial marker), Vimentin, and α-SMA (mesenchymal markers) expression levels normalized to GAPDH. Data are presented as mean ± SEM. n = 4 /group. (G) Immunofluorescence staining for E-cadherin (green), Vimentin (red), and α-SMA (red) in control (PBS), stretch, and HCl-treated cells with or without stretch. Nuclei were counterstained with DAPI (blue). (H) Western blot analysis of PTEN protein expression in control (PBS) and HCl + stretched cells. GAPDH served as a loading control. (I) Quantification of PTEN expression levels normalized to GAPDH. Data are presented as mean ± SEM. n = 4 /group. Comparisons between two groups were performed using independent sample t tests. One-way ANOVA and the Bonferroni post hoc test were used to compare more than two groups. Not significant (ns): *P* > 0.05, *: *P* < 0.05, **: *P* < 0.01, ***: *P* < 0.001, ****: *P* < 0.0001.

Acidic Stimulation and mechanical stretch induced spindle-like morphological changes in BEAS-2B cells, accompanied by increased intercellular spacing (Figure 1A, B). WB revealed significantly decreased E-cadherin (epithelial marker) and increased vimentin/α-SMA (mesenchymal markers) in HCl + stretch vs. control (PBS) cells (Figure 1C-F), consistent with immunofluorescence-confirmed EMT (Figure 1G). PTEN expression was notably reduced in HCl + stretch cells (Figure 1H, I).

**Figure 2:**
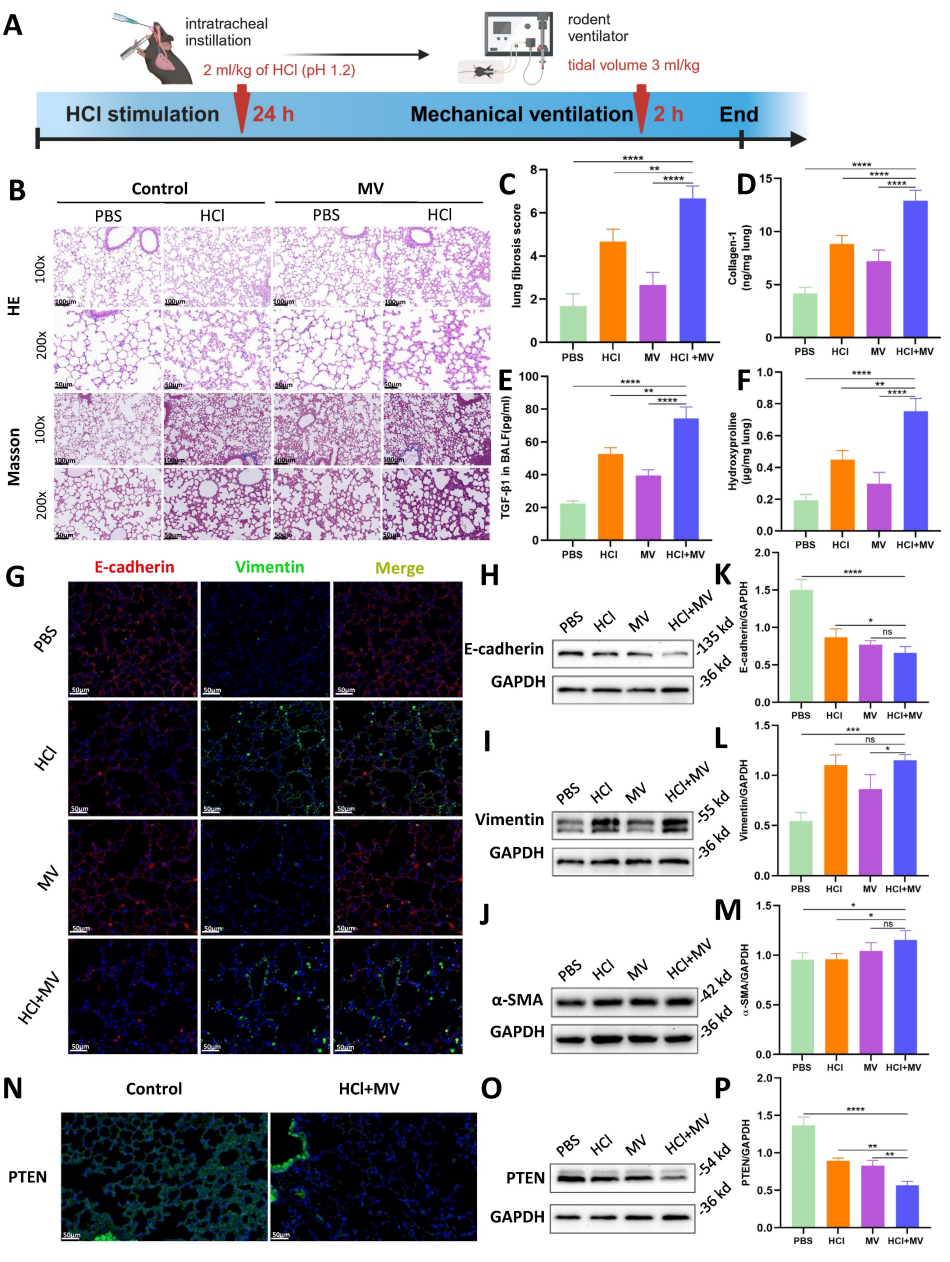
***In vivo* effects of HCl and mechanical ventilation on lung fibrosis and epithelial mesenchymal transition (EMT).** (A) Experimental timeline of "two-hit" (HCl + MV) murine model. Mice were subjected to HCl stimulation followed by mechanical ventilation (MV) for 24 hours and 2 hours, respectively. (B) Hematoxylin and Eosin (HE) and Masson's trichrome staining of lung tissue (14d post-injury) showing inflammatory cell infiltration, alveolar wall thickening, and fibrotic cord formation. Blue: collagen. (C) Ashcroft fibrosis score in control (PBS), MV, and HCl-treated mice with or without MV. Data are presented as mean ± SEM. n = 4 mice /group. (D-F) Quantification of Collagen-I, hydroxyproline in lung tissue, and TGF-β1 levels in bronchoalveolar lavage fluid (BALF). Data are presented as mean ± SEM. n = 4 mice /group. (G) Immunofluorescence staining for E-cadherin (red) and Vimentin (green) in lung sections under different conditions. Nuclei were counterstained with DAPI (blue). (H-J) Western blot analysis of E-cadherin, Vimentin, and α-SMA protein expression in lung tissue under different conditions. (K-M) Quantification of E-cadherin (epithelial marker), Vimentin, and α-SMA (mesenchymal markers) expression levels normalized to GAPDH. Data are presented as mean ± SEM. n = 4 mice /group. (N) Immunofluorescence staining for PTEN (green) in lung sections under different conditions. Nuclei were counterstained with DAPI (blue). (O) Western blot analysis of PTEN protein expression in lung tissue under different conditions. (P) Quantification of PTEN expression levels normalized to GAPDH. Data are presented as mean ± SEM. n = 4 mice /group. One-way ANOVA and the Bonferroni post hoc test were used to compare more than two groups. Not significant (ns): *P* > 0.05, *: *P* < 0.05, **: *P* < 0.01, ***: *P* < 0.001, ****: *P* < 0.0001.

*In vivo* animal experiments found that HCl+MV treatment (Figure 2A) induced severe lung injury, characterized by inflammatory cell infiltration, alveolar wall thickening, and fibrotic cord formation (HE staining; Fig. 2B). Masson's trichrome staining revealed extensive collagen deposition (Fig. 2B,C), supported by elevated collagen I and hydroxyproline levels in lung tissue and increased TGF-β1 in BALF (Fig. 2D-F). EMT was confirmed by decreased E-cadherin and increased vimentin/α-SMA expression (Immunofluorescence/WB; Fig. 2G-M). Notably, PTEN expression was significantly reduced in HCl+MV-treated lungs (Fig. 2N-P).

### Genetic deletion of PTEN protected against MV-PF

**Figure 3:**
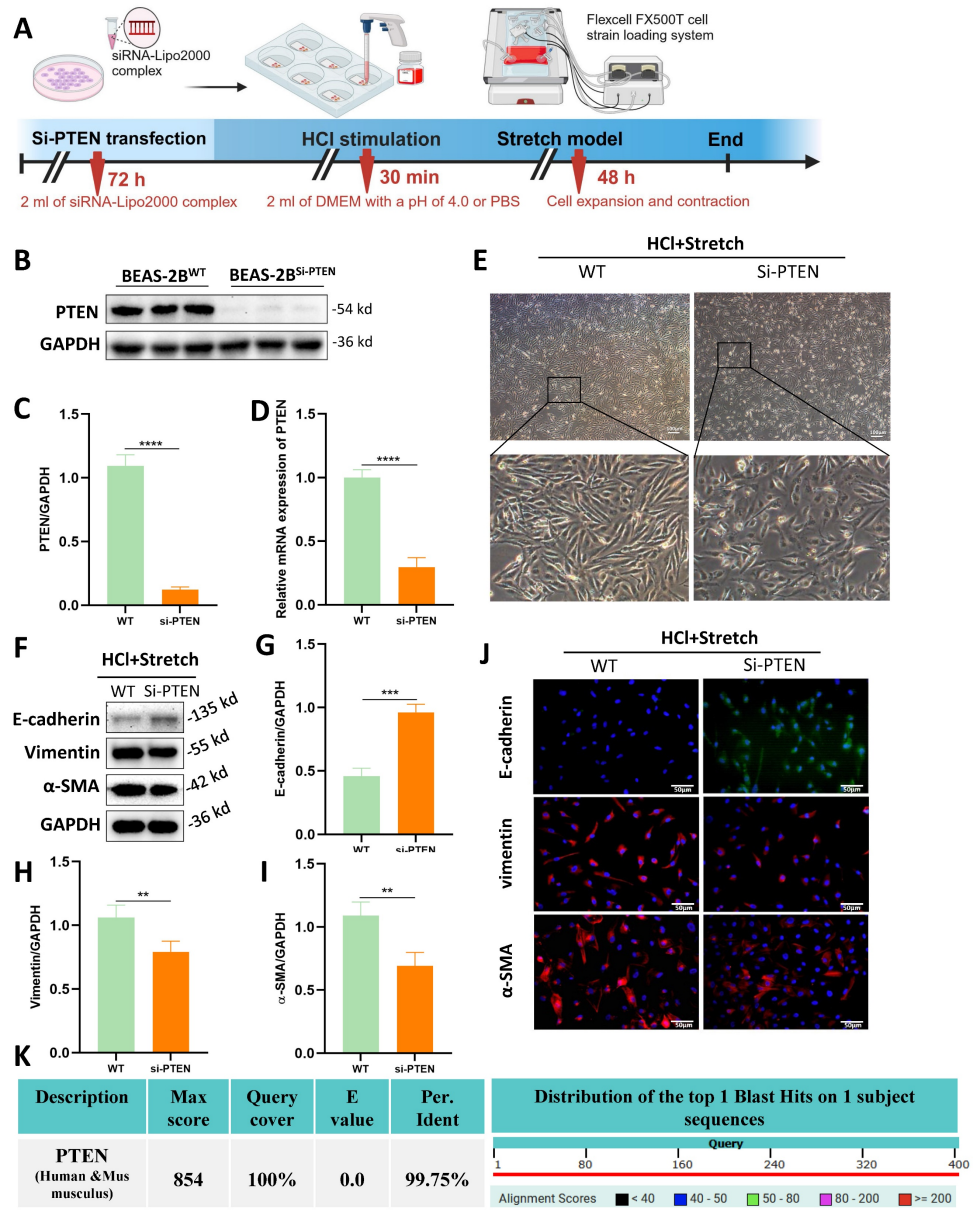
** PTEN knockdown attenuates epithelial mesenchymal transition (EMT) and morphological changes *in vitro*.** (A) Schematic representation of the experimental procedure. BEAS-2B cells were transfected with PTEN siRNA (Si-PTEN), followed by HCl stimulation and mechanical stretch using the Flexcell FX500T cell strain loading system. (B) Western blot analysis of PTEN protein expression in BEAS-2B cells transfected with Si-PTEN. GAPDH served as a loading control. (C) Quantification of PTEN expression levels normalized to GAPDH. Data are presented as mean ± SEM. n = 4 /group. (D) Relative mRNA expression of PTEN in BEAS-2B cells transfected with Si-PTEN. Data are presented as mean ± SEM. n = 4 /group. (E) Representative phase-contrast images showing cell morphology in wild-type (WT) and Si-PTEN transfected cells under HCl + Stretch condition. (F) Western blot analysis of E-cadherin, Vimentin, and α-SMA protein expression in WT and Si-PTEN transfected cells under HCl + Stretch condition. (G-I) Quantification of E-cadherin, Vimentin, α-SMA expression levels normalized to GAPDH. Data are presented as mean ± SEM. n = 4 /group. (J) Immunofluorescence staining for E-cadherin (green), Vimentin (red), and α-SMA (red) in WT and Si-PTEN transfected cells under HCl + Stretch condition. Nuclei were counterstained with DAPI (blue). (K) Sequence alignment of human and mouse PTEN showing 99.75% sequence homology (NCBI alignment score: 854; E-value ≈ 0). The table below the alignment score indicates the percentage identity, and the distribution graph shows the alignment scores of the top 1 Blast Hits on 1 subject sequence. Comparisons between two groups were performed using independent sample t tests. **: *P* < 0.01, ***: *P* < 0.001, ****: *P* < 0.0001.

Although the role of PTEN in chronic IPF is increasingly recognized , its function in acute MV-PF remains unknown. *In vitro*, PTEN small interfering RNA (siRNA) was constructed and transfected into BEAS-2B cells using a transfection reagent (Figure 3A). WB analysis revealed a significant downregulation of PTEN protein expression levels (Figure 3B, C).

RT-qPCR was used to detect PTEN mRNA expression levels in transfected BEAS-2B cells (Figure 3D), showing a significant decrease in PTEN mRNA expression, indicating successful siRNA transfection. Transfection of PTEN siRNA reversed the morphological changes induced by mechanical stretching in BEAS-2B cells (Figure 3E). WB (Figure 3F-I) and immunofluorescence (Figure 3J) revealed that PTEN knockdown improved the EMT phenotype caused by HCl and mechanical stretching in BEAS-2B cells, characterized by increased expression of E-cadherin and decreased expression of Vimentin and α-SMA. Notably, human and mouse PTEN share 99.8% sequence homology (NCBI alignment score: 854; E-value≈0) (Fig. 3K), enabling translational studies in mouse lung tissue to investigate PTEN's role in MV-PF pathogenesis.

**Figure 4:**
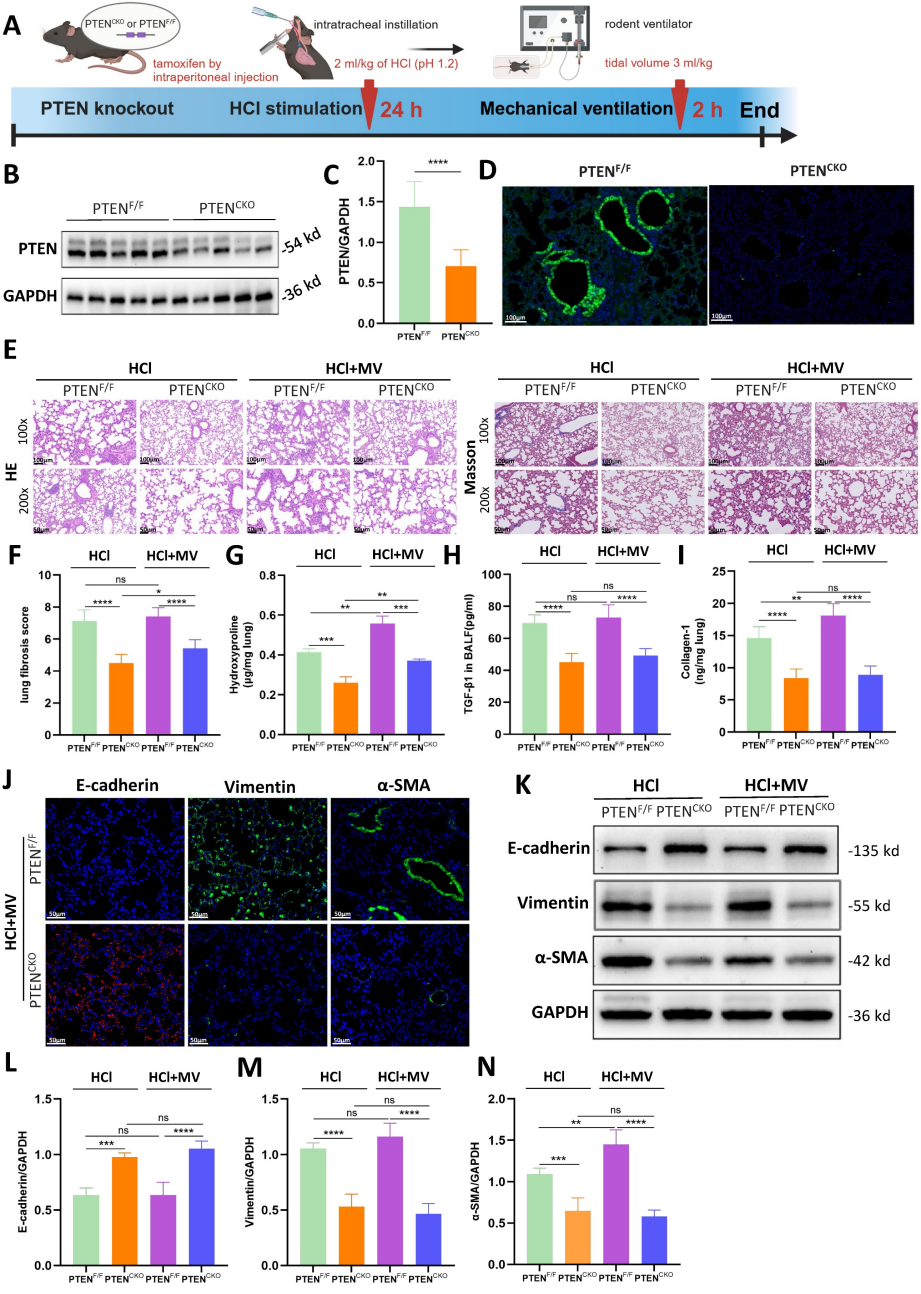
** PTEN knockout alleviates lung injury and epithelial mesenchymal transition (EMT) *in vivo*.** (A) Schematic representation of the experimental procedure. PTEN^CKO^ mice were induced with pulmonary fibrosis using HCl and mechanical ventilation (MV). (B) Western blot analysis of PTEN protein expression in lung tissue from PTEN^F/F^ and PTEN^CKO^ mice. GAPDH served as a loading control. (C) Quantification of PTEN expression levels normalized to GAPDH. Data are presented as mean ± SEM. n = 4 mice/group. (D) Immunofluorescence staining for PTEN (green) in lung sections from PTEN^F/F^ and PTEN^CKO^ mice. Nuclei were counterstained with DAPI (blue). (E) Hematoxylin and Eosin (HE) and Masson's trichrome staining of lung sections showing reduced tissue damage and collagen deposition in PTEN^CKO^ mice compared to PTEN^F/F^ mice after HCl treatment with or without MV. (F) Quantification of Ashcroft fibrosis score in PTEN^CKO^ mice compared to PTEN^F/F^ mice after HCl treatment with or without MV. Data are presented as mean ± SEM. n = 4 mice/group. (G-I) Quantification of Collagen-I, hydroxyproline in lung tissue, and TGF-β1 levels in bronchoalveolar lavage fluid (BALF). Data are presented as mean ± SEM. n = 4 mice/group. (J) Immunofluorescence staining for E-cadherin (red), Vimentin (green), and α-SMA (green) in lung sections from PTEN^CKO^ and PTEN^F/F^ mice under HCl + MV condition. Nuclei were counterstained with DAPI (blue). (K) Western blot analysis of E-cadherin, Vimentin, α-SMA protein expression in lung tissue from PTEN^CKO^ and PTEN^F/F^ mice under different treatment conditions. (L-N) Quantification of E-cadherin, Vimentin, α-SMA expression levels normalized to GAPDH in lung tissue from PTEN^CKO^ and PTEN^F/F^ mice under different treatment conditions. Data are presented as mean ± SEM. n = 4 mice/group. Comparisons between two groups were performed using independent sample t tests. One-way ANOVA and the Bonferroni post hoc test were used to compare more than two groups. Not significant (ns): *P* > 0.05, *: *P* < 0.05, **: *P* < 0.01, ***: *P* < 0.001, ****: *P* < 0.0001.

A lung EC-specific PTEN knockout mouse model using the Cre/Loxp system was established to generate PTEN-flox/Sftpc-cre mice. A comprehensive diagram outlines the experimental workflow starting from the induction of PF in PTEN knockout mice by HCl and MV (Figure 4A). Compared to PTEN^F/F^ mice, PTEN protein expression in the lung tissue of PTEN^CKO^ mice was significantly downregulated as detected by WB (Figure 4B, C). Immunofluorescence results were consistent with WB findings, showing that PTEN was extensively downregulated in the lung tissue of knockout mice, with almost no fluorescence intensity observed in the bronchial ECs (Figure 4D). As shown in Figure 4E, compared to positive control mice, the lung tissue damage in PTEN knockout mice after HCl and HCl+MV was less severe, with only a few fibrous cords and masses, and significantly reduced blue collagen deposition. Quantitative assessment showed PTEN-deficient mice had substantially lower fibrosis scores after combined HCl+MV challenge relative to controls (Figure 4F). Biochemical analyses confirmed these findings, with PTEN knockout animals exhibiting significantly reduced levels of Collagen-I, hydroxyproline, and TGF-β1 in BALF following either HCl alone or HCl+MV exposure (Figure 4G-I). Immunohistochemical and WB analyses further revealed that PTEN deletion reversed the characteristic EMT signature observed in control mice, evidenced by elevated E-cadherin expression and suppressed vimentin and α-SMA levels across HCl and HCl+MV injury conditions (Figure 4J-N).

### scRNA-seq disclosed senescence pathway activation in lung EC following MV-PF

**Figure 5:**
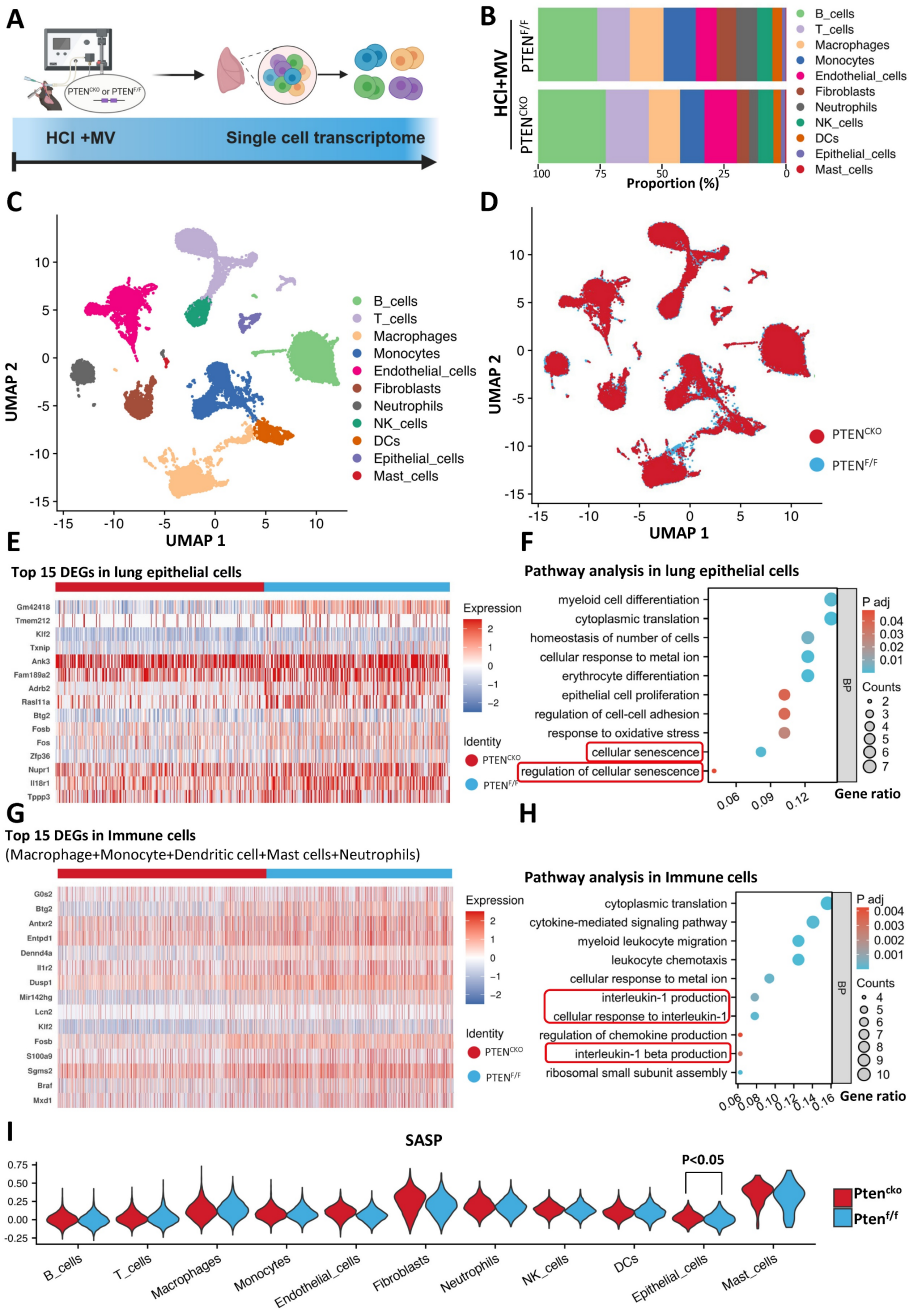
** Single-cell transcriptomics implicates senescence pathways in PTEN-regulated mechanical ventilation-associated pulmonary fibrosis.** (A) Schematic representation of the single-cell RNA sequencing (scRNA-seq) workflow. Following HCl and mechanical ventilation (MV), lung tissues were extracted, single cells were isolated, and next-generation sequencing was performed using the 10x Genomics platform. (B) Proportion of different cell types in lung tissue from PTEN^F/F^ and PTEN^CKO^ (knockout) mice under HCl+MV condition. (C) Uniform Manifold Approximation and Projection (UMAP) plot showing distinct cell populations in lung tissue from mice under HCl+MV condition. (D) UMAP plot comparing PTEN^F/F^ and PTEN^CKO^ mice under HCl+MV condition, highlighting changes in cellular distribution patterns. (E) Heatmap of the top 15 differentially expressed genes (DEGs) in lung epithelial cells (ECs) following PTEN knockout. (F) Pathway analysis in lung ECs showing significant enrichment in pathways related to cellular senescence. (G) Heatmap of the top 15 DEGs in immune cells (macrophages, monocytes, dendritic cells, mast cells, and neutrophils) following PTEN knockout. (H) Pathway analysis in immune cells showing significant enrichment in pathways related to interleukin-1 and interleukin-1 β signaling (cellular senescence-related pathways). (I) Senescence-associated secretory phenotype (SASP) scores in 11 major lung cell types (AddModuleScore), with the highest scores observed in mast cells and fibroblasts. Differences in SASP scores were observed only in lung ECs, indicating a protective effect of PTEN knockout on MV-PF through downregulation of cellular senescence in lung ECs. Comparisons between two groups were performed using independent sample t tests.

These findings establish PTEN knockout as pivotal for reversing MV-PF. To elucidate how PTEN promotes MV-PF, we utilized scRNA-seq to reveal changes in different lung cells in MV-PF model following PTEN knockout (Figure 5A). The measurement process includes lung tissue extraction, single-cell isolation, next-generation sequencing analysis using the 10x Genomics platform, detailed bioinformatics analysis, and subsequent validation of results. Using Uniform Manifold Approximation and Projection (UMAP), we identified cell populations based on the expression of known cell type-specific marker genes. Through these approaches, we delineated 11 distinct lung cell populations - including B cells, T cells, phagocytes, monocytes, endothelial cells, fibroblasts, neutrophils, Natural Killer (NK) cells, Dendritic cells (DCs), ECs, and mast cells (Figure 5B, C). Comparative analysis revealed PTEN knockout significantly altered cellular distribution patterns in the lung microenvironment (Figure 5D), demonstrating its broad impact on pulmonary cell ecology during fibrotic progression.

Heatmap analysis further confirmed the presence of numerous differentially expressed genes (DEGs) in lung tissues during MV-PF injury after PTEN knockout, particularly within lung ECs and the pulmonary immune cell microenvironment. Figure 5E shows the top 15 DEGs in MV-PF lung ECs following PTEN knockout. Additionally, GO pathways related to cellular oxidative damage and response to mechanical stimulation were significantly enriched. Notably, the cellular senescence signaling pathway was positively regulated in PTEN^F/F^ mice (Figure 5F). DEGs analysis and pathway enrichment analysis of pulmonary immune cell clusters revealed significant upregulation of senescence-associated secretory phenotype (SASP) pathways, such as IL-1 and IL-1β (Figures 5G, H), suggesting that PTEN expression activation can promote cellular senescence in the pulmonary microenvironment.

To further analyze the changes in SASP during MV-PF after PTEN knockout, we used the AddModuleScore function to score and visualize the senescence gene set in single cells. To systematically identify SASP-related genes, we conducted a PubMed search using the keywords 'Senescence-Associated Secretory Phenotype' and 'Review,' limiting results to studies published between 2017 and 2024 for relevance. This strategy focused on review articles that extensively covered SASP-related genes in both normal and disease states. From three selected reviews 24-26, we compiled a list of SASP genes, yielding 81 unique entries after deduplication (see Supplementary Table S3 for the complete list). Figures 5I shows the scores of the SASP gene set in the 11 major cell types of lung tissue, with the highest scores in mast cells and fibroblasts. Furthermore, differences in SASP scores were observed only in lung EC, indicating that PTEN knockout exerts a protective effect on MV-PF through the downregulating of the cellular senescence in lung EC.

### PTEN knockdown ameliorated fibrosis-associated senescence

**Figure 6:**
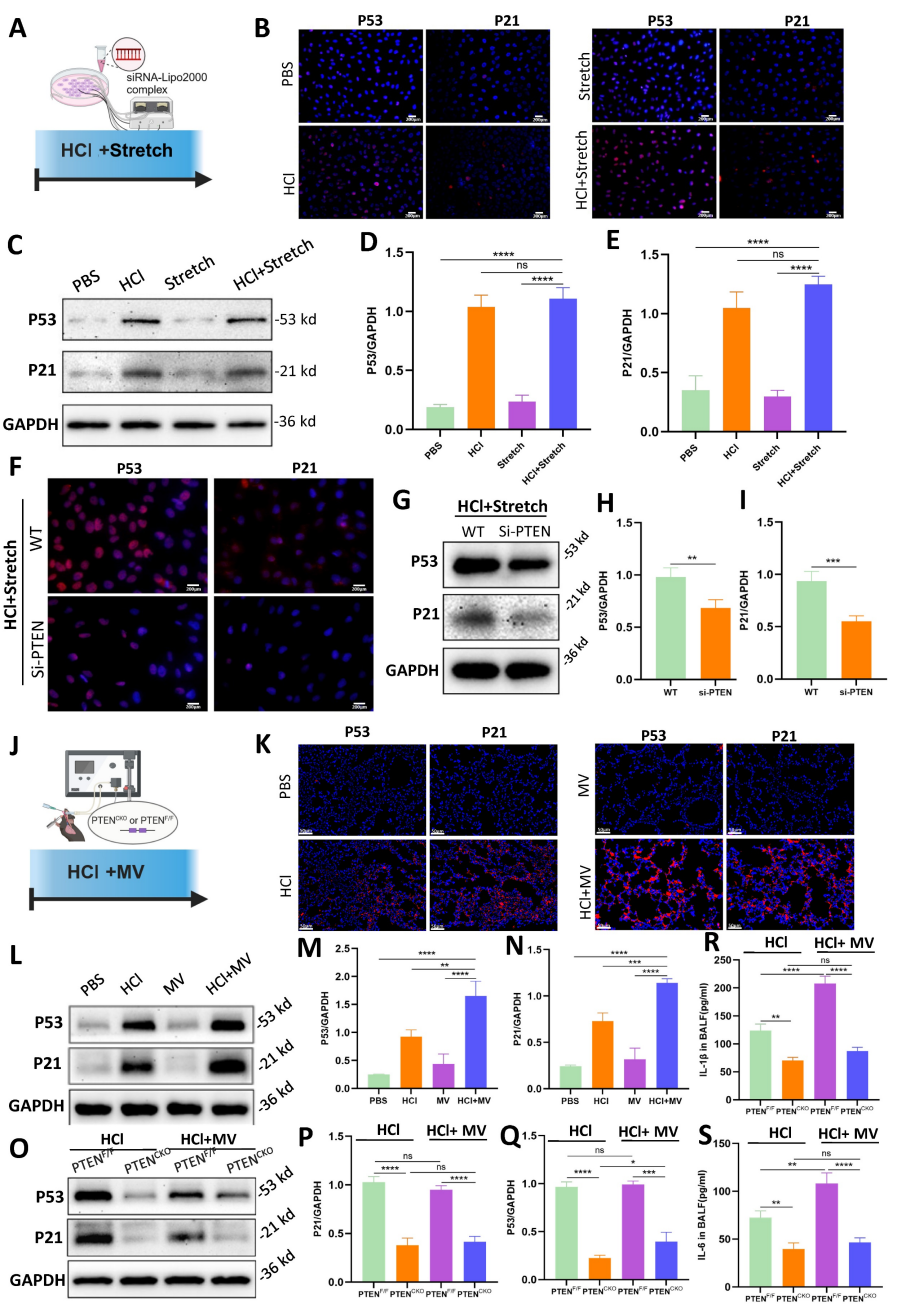
** PTEN knockdown suppresses cell senescence *in vitro* and *in vivo*.** (A) Schematic representation of the *in vitro* experimental procedure. BEAS-2B cells were transfected with or without PTEN siRNA and subjected to HCl + stretch. (B) Immunofluorescence staining for P53 (red) and P21 (red) in BEAS-2B cells under different conditions. Nuclei were counterstained with DAPI (blue). (C) Western blot analysis of P53 and P21 protein expression in BEAS-2B cells. (D-E) Quantification of P53 and P21 expression levels normalized to GAPDH. Data are presented as mean ± SEM. n = 4 /group. (F) Immunofluorescence staining for P53 (red) and P21 (red) in BEAS-2B cells following PTEN knockdown under HCl + Stretch condition. Nuclei were counterstained with DAPI (blue). (G) Western blot analysis of P53 and P21 protein expression in BEAS-2B cells following PTEN knockdown under HCl + Stretch condition. (H-I) Quantification of P53 and P21 expression levels normalized to GAPDH. Data are presented as mean ± SEM. n = 4 /group. (J) Schematic representation of the *in vivo* experimental procedure. Mice were subjected to HCl stimulation and mechanical ventilation (MV). (K) Immunofluorescence staining for P53 (red) and P21 (red) in lung tissue from mice under different conditions. Nuclei were counterstained with DAPI (blue). (L) Western blot analysis of P53 and P21 protein expression in lung tissue from mice under different conditions. (M-N) Quantification of P53 and P21 expression levels normalized to GAPDH. Data are presented as mean ± SEM. n = 4 mice/group. (O) Western blot analysis of P53 and P21 protein expression in lung tissue from PTEN^F/F^ and PTEN^CKO^ mice under HCl with or without MV conditions. (P-Q) Quantification of P53 and P21 expression levels normalized to GAPDH. Data are presented as mean ± SEM. n = 4 mice/group. (R) Quantification of IL-1β levels in bronchoalveolar lavage fluid (BALF) from mice under different conditions. Data are presented as mean ± SEM. n = 4 mice/group. (S) Quantification of IL-6 levels in BALF from mice under different conditions. Data are presented as mean ± SEM. n = 4 mice/group. Comparisons between two groups were performed using independent sample t tests. One-way ANOVA and the Bonferroni post hoc test were used to compare more than two groups. Not significant (ns): *P* > 0.05, *: *P* < 0.05, **: *P* < 0.01, ***: *P* < 0.001, ****: *P* < 0.0001.

*In vitro* cellular experiments (Figure 6A), compared to the PBS control group, the HCl + stretch groups exhibited a marked increase in P53 and P21 fluorescence intensity (Figure 6B). WB analysis further confirmed that P53 and P21 protein expression was significantly elevated in the HCl + stretch groups compared to normal cells in the control group (Figure 6C-E). Additionally, both immunofluorescence and WB analyses revealed a significant decrease in P53 and P21 expression in BEAS-2B cells following PTEN knockdown (Figure 6F-I).

*In vivo* experiments (Figure 6J), compared to the PBS control group, mice treated with HCl + MV exhibited a significant increase in P53 and P21 fluorescence intensity in lung tissue (Figure 6K). WB analysis revealed that P53 and P21 expression increased in mice from the HCl + MV group compared to the control group (Figure 6L-N). *In vivo* PTEN knockout experiments showed that compared to the positive control group, the expression of P53 and P21 in the lung tissue of the PTEN-knockout mice group was significantly reduced (Figure 6O-Q). Furthermore, the expression of IL-1β and IL-6 in the BALF of mice was also significantly reduced in the PTEN-knockout group (Figure 6R, S).

### PTEN regulated P53-mediated cellular senescence in MV-PF

**Figure 7:**
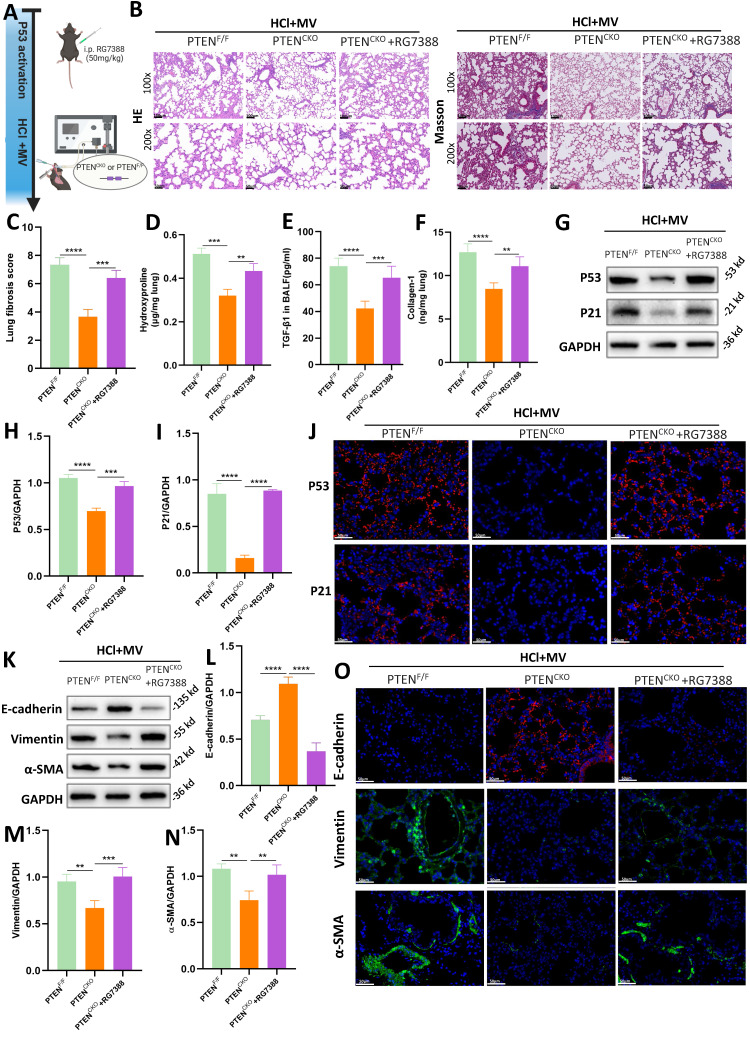
** PTEN regulates epithelial mesenchymal transition (EMT) via P53-senescence axis.** (A) Schematic representation of the experimental design involving P53 activation using the MDM2-P53 complex inhibitor RG7388 in PTEN^CKO^ or PTEN^F/F^ mice subjected to HCl and mechanical ventilation (MV). (B) Hematoxylin and Eosin (HE) and Masson's trichrome staining of lung sections from PTEN^F/F^, PTEN^CKO^, and PTEN^CKO^ + RG7388 mice under HCl+MV condition. (C) Quantification of Ashcroft fibrosis score in PTEN^F/F^, PTEN^CKO^, and PTEN^CKO^ + RG7388 mice under HCl+MV condition. Data are presented as mean ± SEM. n = 4 mice/group. (D-F) Quantification of Collagen-I, hydroxyproline in lung tissue, and TGF-β1 levels in bronchoalveolar lavage fluid (BALF) under HCl+MV condition. Data are presented as mean ± SEM. n = 4 mice/group. (G) Western blot analysis of P53 and P21 protein expression in lung tissue from PTEN^F/F^, PTEN^CKO^, and PTEN^CKO^ + RG7388 mice under HCl+MV condition. (H-I) Quantification of P21 and P53 expression levels normalized to GAPDH. Data are presented as mean ± SEM. n = 4 mice/group. (J) Immunofluorescence staining for P53 (red) and P21 (red) in lung sections from PTEN^F/F^, PTEN^CKO^, and PTEN^CKO^ + RG7388 mice under HCl+MV condition. Nuclei were counterstained with DAPI (blue). (K) Western blot analysis of E-cadherin, Vimentin and α-SMA protein expression in lung tissue from PTEN^F/F^, PTEN^CKO^, and PTEN^CKO^ + RG7388 mice under HCl+MV condition. (L-N) Quantification of E-cadherin, Vimentin and α-SMA expression levels normalized to GAPDH. Data are presented as mean ± SEM. n = 4 mice/group. (O) Immunofluorescence staining for E-cadherin (red), Vimentin (green), and α-SMA (green) in lung sections from PTEN^F/F^, PTEN^CKO^, and PTEN^CKO^ + RG7388 mice under HCl+MV condition. Nuclei were counterstained with DAPI (blue). One-way ANOVA and the Bonferroni post hoc test were used to compare more than two groups. **: *P* < 0.01, ***: *P* < 0.001, ****: *P* < 0.0001.

RG7388 is an inhibitor of the MDM2-P53 complex, which reduces the degradation of P53 in the nucleus and increases its levels (Figure 7A). As shown in Figure 7B, histological staining with HE and Masson's trichrome revealed that in PTEN^CKO^ mice treated with HCl and MV, those administered RG7388 exhibited significantly more severe lung damage compared to the untreated PTEN^CKO^ group, with damage levels similar to those in PTEN^F/F^ mice. This included marked thickening of the alveolar walls, structural disruption, and extensive collagen deposition. Compared to PTEN^CKO^ mice, the fibrosis score in PTEN^CKO^ mice treated with RG7388 was significantly higher (Figure 7C). Compared to PTEN^CKO^ mice not treated with the inhibitor, those treated with RG7388 showed significant increases in Collagen-I, hydroxyproline, and TGF-β1 (Figure 7D-F). These results indicate that upregulation of P53 can exacerbate the fibrotic damage caused by HCl + MV in PTEN^CKO^ mice. Collectively, these findings suggest that PTEN knockout can mitigate the fibrotic damage of lung tissue in mice, but this protective effect is reversed upon P53 upregulation with RG7388, leading to increased lung damage.

As illustrated in Figures 7G-I and J, WB and immunofluorescence analyses revealed that in PTEN^CKO^ mice treated with intraperitoneal injections of RG7388, there was a significant increase in the expression of P53 and P21 in lung tissue. This indicates that RG7388 effectively exerts its action, promoting the expression of cellular senescence markers. Furthermore, as shown in Figures 7K-O, the upregulation of P53 using the inhibitor RG7388 exacerbated EMT in PTEN^CKO^ mice.

## Discussion

**Figure 8:**
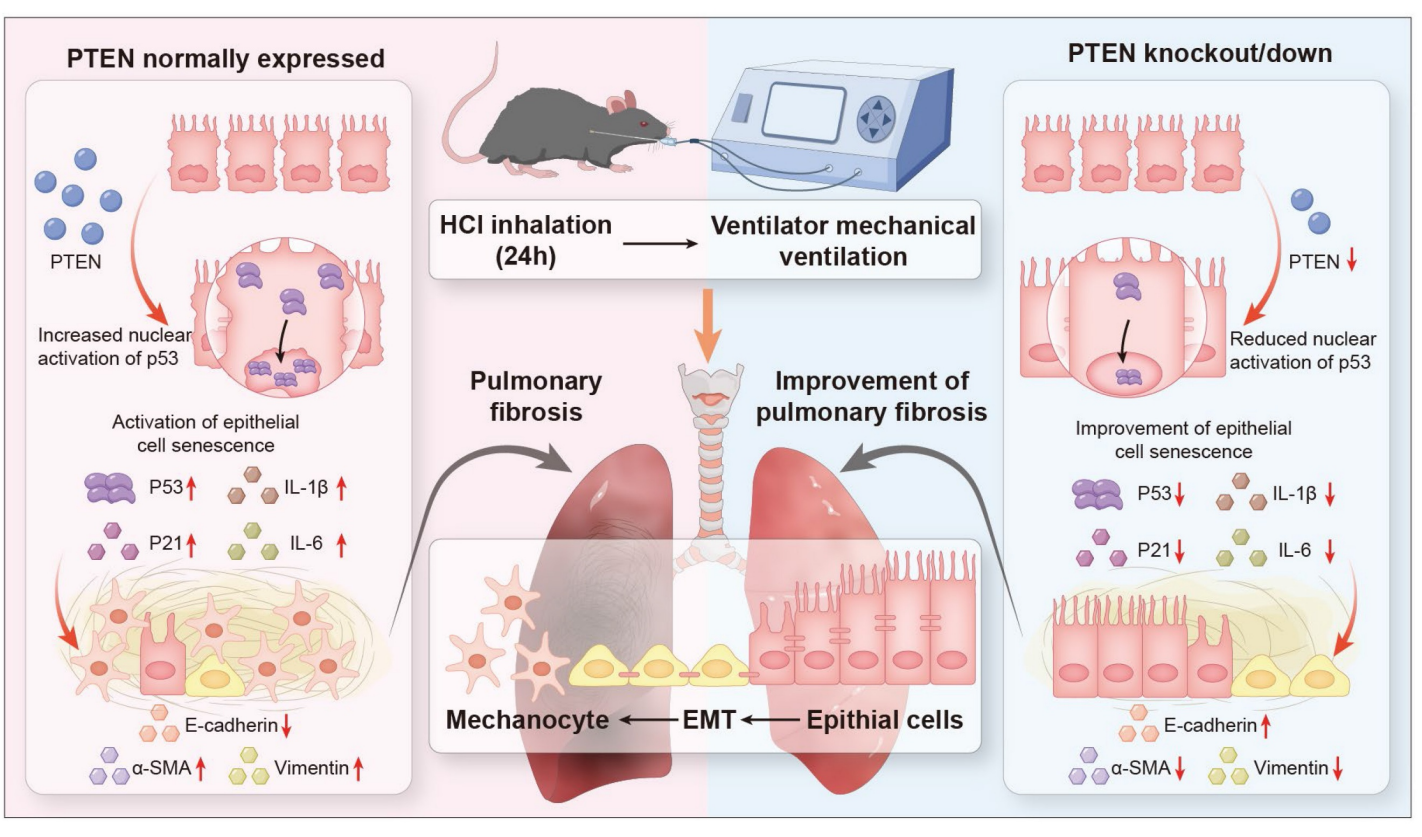
** Schematic representation of the role of PTEN in mediating EMT and cellular senescence in MV-PF.** Proposed model: PTEN upregulation in mechanical ventilation-associated pulmonary fibrosis promotes P53-mediated epithial cell senescence, driving epithelial mesenchymal transition (EMT) and fibrosis. PTEN knockout or inhibition ameliorates this cascade.

The progression of PF in the context of MV and acid aspiration, underscores the need for a deeper understanding of the underlying mechanisms. Current research predominantly focused on the roles of EMT and cellular senescence, yet the interplay between these processes and their upstream regulators remains underexplored, particularly in MV-PF. The findings reveal that PTEN, previously recognized as an anti-fibrotic molecule, may instead mediate EMT through the P53 cell senescence pathway in MV-PF. By integrating *in vitro* and *in vivo* approaches, using PTEN gene editing and RNA interference techniques, single-cell omics detection, etc., this research contributes valuable insights into the molecular dynamics of MV-PF and the pathological progression towards fibrosis, emphasizing the clinical relevance of PTEN in therapeutic strategies. In summary, our findings indicate that downregulating PTEN expression can inhibit P53-mediated senescence of lung ECs, thereby ameliorating EMT and MV-PF (Figure 8).

IPF carries a poor prognosis due to irreversible ECM deposition and lung tissue destruction 27. Even for acute exacerbations of IPF, current anti-inflammatory agents and immunosuppressants fail to improve survival rates 28. For MV-associated fibrosis, prolonged MV exposure (e.g., in COVID-19 patients) may induce accelerated EMT, contributing to treatment-resistant fibrosis 29. However, experimental evidence suggests that acute-onset MV-PF may be partially reversible through early intervention 16. Protective ventilation strategies reducing lung stretch have improved outcomes by minimizing ventilator-induced injury 30, highlighting the need for targeted therapies during this potentially reversible phase.

PTEN serves as an antifibrotic mediator in IPF by stabilizing the alveolar basement membrane and inhibiting fibroblast proliferation, differentiation, and collagen secretion 20. Interestingly, our preliminary data suggest differential PTEN signaling patterns in MV-PF compared to traditional chronic PF, highlighting the necessity for mechanistic investigations into PTEN-regulated pathways. Specifically, PTEN downregulation was observed in both *in vitro* and *in vivo* MV-PF models. However, this downregulation may not exacerbate fibrotic progression; instead, it could reflect a self-protective compensatory mechanism in which reduced PTEN expression paradoxically attenuates fibrosis. Corroborating this hypothesis, prior studies report that PTEN inhibition ameliorates cardiac fibrosis through M2 macrophage polarization 21. To clarify PTEN's role in MV-PF, we employed PTEN knockdown or knockout strategies across *in vivo* and vitro experimentals. Remarkably, these interventions reduced collagen deposition and significantly alleviated EMT, indicating that targeted PTEN might hold therapeutic potential for MV-driven fibrotic injury.

To explore the molecular mechanisms by which PTEN knockout alleviates EMT and MV-PF, we employed single-cell sequencing to generate a cellular atlas of MV-PF and to examine changes in the cellular microenvironment post-PTEN knockout. Our results indicated that while PTEN knockout had minimal impact on cell clustering and quantity, it led to a series of DEGs within lung ECs and immune cell clusters, which were enriched in pathways related to cellular senescence. SASP scoring of the major cell clusters showed differences primarily in lung ECs. Senescent cells, through their secretome known as the SASP, are a major driver of fibrotic diseases 31,32.

Senescence of alveolar type 2 ECs is sufficient to drive progressive fibrotic injury 33. Early targeting senescence-related pathways in lung ECs, particularly type II ECs, represents a promising therapeutic approach for preventing IPF. For instance, researchers have found that YAP1, by targeting Prdx3, can improve mitochondrial function, inhibit the senescence of alveolar type II ECs, and thereby alleviate IPF 34. Under physiological conditions, alveolar type II ECs in the injured lung activate self-repair programs, promoting their own proliferation and differentiation into alveolar type I ECs to maintain alveolar epithelial homeostasis 35. Persistent lung injury can lead to the senescence of alveolar type II ECs and impaired lung repair, ultimately resulting in cronic PF 33. However, the role and initiation mechanisms of lung ECs senescence in MV-PF have not yet been fully elucidated. Sftpc is a marker for alveolar type II ECs. In this study, PTEN^FL/FL^ mice were crossed with Sftpc-Cre^ERT^ mice to establish a conditional PTEN knockout mouse model, primarily targeting the deletion of PTEN gene expression in alveolar type II ECs. The results showed an increased expression of senescence markers in response to HCl-MV, which diminishes upon PTEN knockout, suggests that PTEN may regulate EMT through the SASP that contributes to the fibrotic process.

Moreover, the interaction between the PTEN and P53 signaling pathways is particularly noteworthy. The downregulation of P53, a well-known regulator of cellular senescence 36,37, in the absence of PTEN suggests a mechanistic link between PTEN deficiency and the alleviation of senescence-related pathways. The observation that P53 activation can reverse the protective effects of PTEN knockout further supports this relationship, indicating that PTEN may influence fibrosis and EMT by modulating P53-mediated cellular responses. In cancers, concurrent loss of PTEN and P53 paradoxically induces tumor development and exacerbates poor prognosis, as seen in gliomas 38 and triple-negative breast cancer 39. However, recent studies have unveiled a synergistic role of PTEN and P53 in DNA damage and cellular senescence. For instance, recent research reports that both P53 and PTEN are key cell fate regulators and that they have a synergistic role in the DNA damage response. Controlling the dynamics of PTEN and P53 may be a feasible strategy 40. Studies have also revealed the significant roles of the PTEN and P53 signaling pathways in the oxidative stress and replicative senescence of mesenchymal stem cells 41.

Our findings position PTEN as a potential therapeutic target for acute MV-PF patients. The temporal specificity of PTEN's pro-fibrotic action suggests that inhibition strategies may require precise timing—early in MV-PF. In summary, the interplay between PTEN knockout and the resultant senescence phenotypic changes in lung EC provides a compelling framework for understanding the molecular underpinnings of PF. Targeting these pathways may offer novel therapeutic avenues to address the challenges posed by MV-related fibrotic injuries.

## Supplementary Material

Supplementary methods and tables.

## Abbreviations

ALI/ARDS: Acute lung injury/acute respiratory distress syndrome
ECs: epithelial cells
PF: pulmonary fibrosis
ILD: interstitial lung disease
ECM: extracellular matrix
MV: Mechanical ventilation
IPF: idiopathic pulmonary fibrosis
EMT: epithelial-mesenchymal transition
PTEN: Phosphatase and tensin homolog deleted on chromosome 10
MV-PF: MV-associated pulmonary fibrosis
ATCC: American Type Culture Collection
MEM: Minimal Essential Medium
DMEM: Dulbecco's Modified Eagle Medium
HE: Hematoxylin and Eosin
WB: Western blot
IF: Imunofluorescence
ELISA: Enzyme-linked-immunosorbent serologic assay
BALF: bronchoalveolar lavage fluid
STR: Short Tandem Repeat
UMAP: Using Uniform Manifold Approximation and Projection
NK: Natural Killer
DCs: Dendritic cells
DEGs: differentially expressed genes
SASP: senescence-associated secretory phenotype
