# Supplementary material for: PTEN-mediated senescence of lung epithelial cells drives ventilator-induced pulmonary fibrosis
Source: Theranostics. 2025 Jul 25;15(16):8360–76. doi: 10.7150/thno.117523 (PMC12374584; doi:10.7150/thno.117523)
Supplement: Supplementary file 1 — Supplementary methods and tables. [file thnov15p8360s1.pdf]

Supplementary Online Content

PTEN-Mediated Senescence of Lung Epithelial Cells Drives Ventilator-Induced Pulmonary Fibrosis

Mengyu Li et al.

Supplementary Methods 2

1. Antibodies for western blot assay and imunofluorescence assay 2

2. The primer sequences of quantitative real-time PCR 3

3. A list of SASP genes, yielding 81 unique entries from three review literature (17,26,27) ... 4

Supplementary Methods

1. Antibodies for western blot assay and imunofluorescence assay

Antibodies are listed in Supplementary Table S1.

Table S1. The Primary antibody

Protein	Antibody
α-SMA	Abcam, Wuhan, China
E-cadherin	CST， USA
Vimentin	Santa Cruz, USA
P53	CST, USA
P21	Santa Cruz, USA
PTEN	CST, USA
GAPDH	Proteintech, Wuhan, China
HRP Goat-anti-Mouse IgG	Proteintech, Wuhan, China
HRP Goat-anti-Rabbit IgG	Proteintech, Wuhan, China
Dylight594 Goat-Anti-Mouse	Abbikin, Wuhan, China
Dylignt488 Goat-Anti-Mouse	Abbikin, Wuhan, China
Dylight488 Goat-Anti-Rabbi	Abbikin, Wuhan, China
Dylignt488 Goat-Anti-Mouse IgG	Abbikin, Wuhan, China
Dylight488 Goat-Anti-Rabbit IgG	Abbikin, Wuhan, China

2. The primer sequences of quantitative real-time PCR

The primer sequences are detailed in Table S2.

Table S2. The primer sequences

Gene	The primer sequences（5` -3` ）	
PTEN	Forward	TGGATTCGACTTAGACTTGACCT
	Reverse	GGTGGGTTATGGTCTTCAAAAGG
GAPDH	Forward	TGTGGGCATCAATGGATTTGG
	Reverse	ACACCATGTATTCCGGGTCAAT
β-actin	Forward	AATTGCTTCCACAATCCGAAC
	Reverse	TGCTGTCACCTTCACCGTTC

3. A list of SASP genes, yielding 81 unique entries from three review literature (17,26,27)

Table S3. List of 81 genes of SASP related gene signature from three review literature (17,26,27)

Gene lists	
1	1L-15
2	AREG
3	ATM
4	AUF1
5	B2M
6	C/EBPβ
7	CCL2
8	CCL5
9	CDKN2a
10	CEBPB
11	Cgas
12	CHK2
13	CM-CSF
14	CSF1
15	CTCF
16	CTGF
17	CXCL1
18	CXCL12
19	CXCL2
20	CXCL5
21	DDR
22	DEP1
23	DPP4
24	E2F
25	EZH2
26	FOXA1
27	GATA4
28	H2AFY
29	H2AJ
30	HAPLN1
31	HDAC
32	HGF
33	HMGB1
34	HMGB2
35	HSP27
36	ICAM1

37	IFNs
38	IL-1
39	IL-10
40	IL-13
41	IL6
42	IL-8
43	IRAK1
44	JAK2
45	Klf4
46	M-CSF
47	MK2
48	MLL1
49	MMP1
50	MMP2
51	MTOR
52	Myc
53	NBS1
54	ND
55	NFKB1
56	NFKB2
57	NKG2D
58	NOTCH1
59	NOTCH3
60	OCT
61	p16INK4a
62	p38MAPK
63	p53
64	PAR1
65	PDGF-AA
66	PGE2
67	RB
68	Sfrp2
69	SHP2
70	SIRT1
71	SOX2
72	STAT3
73	sting
74	TGFB
75	TNF

76	TNFa
77	TREX1
78	uPAR
79	VEGF
80	WNT16B
81	β-catenin
